# Data for the characterization of the HSP70 family during osmotic stress in banana, a non-model crop

**DOI:** 10.1016/j.dib.2015.01.008

**Published:** 2015-02-13

**Authors:** Anne-Catherine Vanhove, Wesley Vermaelen, Alberto Cenci, Rony Swennen, Sebastien C. Carpentier

**Affiliations:** a Laboratory of Tropical Crop Improvement, Division of Crop Biotechnics, KU Leuven, Leuven, Belgium; b Facility for Systems Biology based Mass Spectrometry (SYBIOMA), KU Leuven, Leuven, Belgium; c Bioversity International, Montpeller, France; d Bioversity International, Leuven, Belgium; e International Institute of Tropical Agriculture, Arusha, Tanzania

**Keywords:** Non-model plant, Protein family, HSP70, Allelic variant, Paralog

## Abstract

Here, we present the data from an in-depth analysis of the HSP70 family in the non-model banana during osmotic stress [1]. First, a manual curation of HSP70 sequences from the banana genome was performed and updated on the Musa hub http://banana-genome.cirad.fr/. These curated protein sequences were then introduced into our in-house Mascot database for an in-depth look at the HSP70 protein profiles in banana meristem cultures and roots during osmotic stress. A 2D-DIGE LC MS/MS approach was chosen to identify and quantify the different paralogs and allelic variants in the HSP70 spots.

**Specifications table**

**Table:** 

Subject area	Biology

More specific subject area	Plant proteomics, HSP70 family
Type of data	Tables, text files, figures
How data was acquired	2D-DIGE and mass spectrometry (Q Exactive Orbitrap)
Data format	Analyzed
Experimental factors	Meristem cultures and plant roots were exposed to osmotic stress
Experimental features	Meristem and root proteomes were separated using 2D-DIGE and proteins belonging to the HSP70 family were identified using LC MS/MS
Data source location	Leuven, Belgium
Data accessibility	Within this article

**Value of the data**

• Data provides an overview of the identification and quantification of the HSP70 family in a non-model plant with special attention to osmotic stress.
• Manual curation of the banana HSP70 sequences was performed as correct structural annotation of a sequenced genome is essential prior to proteomic analysis.
• The proteomics approach using 2DE and LC MS/MS was a deciding factor in the successful identification and quantification of the HSP70 paralogs and allelic variants.

## 1 Experimental design, materials and methods

### 1.1 Analysis of the *Musa* HSP70 family

**Fig. 1:**
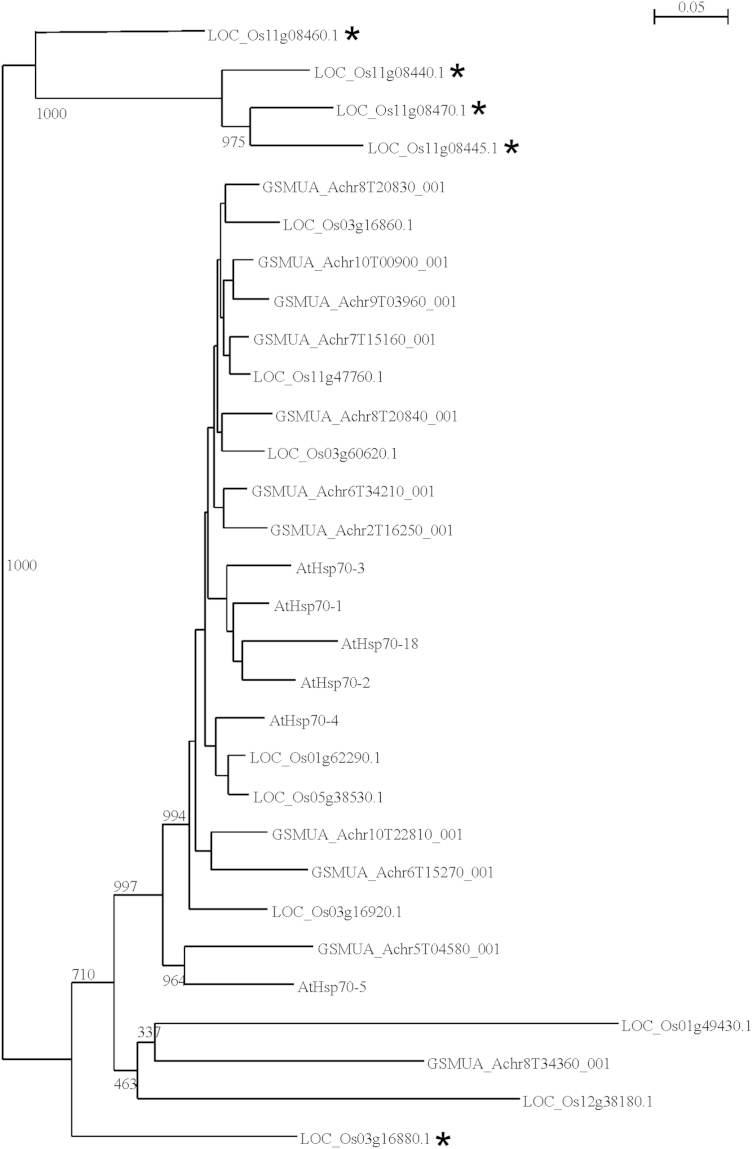
Phylogenetic relationship between all curated cytoplasmic HSP70 protein sequences of the Musa A genome (GSMUA_Achr) and the cytoplasmic HSP70s of rice (LOC_Os) and Arabidopsis (AtHsp). Sequences were aligned using ClustalX and a neighbor-joining tree was constructed with 1000 replicate bootstrap. The bootstrap values between major groups are indicated. The non classical cytoplasmic rice HSP70 accessions have been indicated with ⁎.

*Musa* HSP70 nucleotide and protein sequences were obtained from GreenPhyl and the Banana Genome Hub [2–4]. Since many HSP70 genes were incorrectly predicted, all HSP70 predicted from the Acuminata A genome sequences were manually curated as well as the cytoplasmic and luminal HSP70 sequences predicted from the Balbisiana B genome. The manually curated B genome cytoplasmic and luminal protein sequences can be found in Supplementary file 1. The manually curated A genome protein sequences are available at the Banana Genome Hub. Cytoplasmic HSP70 from rice and *Arabidopsis thaliana* were retrieved from Greenphyl with the accessions as described by Jung et al. [5]. Alignments of protein sequences were created using the ClustalX 2.1 software [6]. An alignment of the main cytoplasmic HSP70 isoforms identified later in this manuscript can be found in Supplementary file 2. Phylogenetic trees were constructed *via* ClustalX 2.1 using the neighbor-joining algorithm with 1000 replicate bootstrap tests. Trees were visualized with njplot [7]. The phylogenetic relationship between all curated cytoplasmic HSP70 protein sequences of the Musa A genome (GSMUA_Achr) and the cytoplasmic HSP70s of rice (LOC_Os) and Arabidopsis (AtHsp) can be found in Fig. 1.

### 1.2 *In vitro* meristem stress tests

*In vitro* plants of the selected variety Cachaco (ABB, ITC 0643) were supplied by the International Transit Centre of Bioversity International. Multiple shoot meristem cultures were initiated as described by Strosse et al. [8] and maintained on the standard control medium (MS medium supplemented with benzylaminopurine). All cultures were kept in dark at 25–27 °C. A stress test was started by adding 0.31 M sucrose to the standard medium. Tissue samples of stressed meristem cultures were taken and frozen after 0, 1, 4 and 14 days. All samples were stored at −80 °C.

### 1.3 Plant root stress test

*In vitro* plants of the selected variety Cachaco (ABB, ITC 0643) were supplied by the International Transit Centre of Bioversity International. The plants were grown in a phytotron (Sanyo, MLR-351H). The humidity and temperature were kept constant at 75% and 25 °C respectively. A 12 h/12 h light/dark period with an average light intensity of 183±29 µmol photons m^−2^ s^−1^ was maintained throughout the experiment. To apply a physiological more relevant osmotic stress we lowered the osmotic concentration to apply moderate stress during which plants suffered a reduction in growth but not a full growth stop and switched to the non-metabolizable sorbitol. After five weeks an osmotic stress test was started by adding 0.21 M sorbitol to the MSR medium (MSR medium according to Voets et al. [9]). Root samples of control plants were taken and frozen in liquid nitrogen at the start of the experiment and after 4 days. Root samples of sorbitol stressed plants were taken and frozen after 0, 1, 4 and 14 days. All samples were stored at −80 °C.

### 1.4 Proteomics

**Fig. 2:**
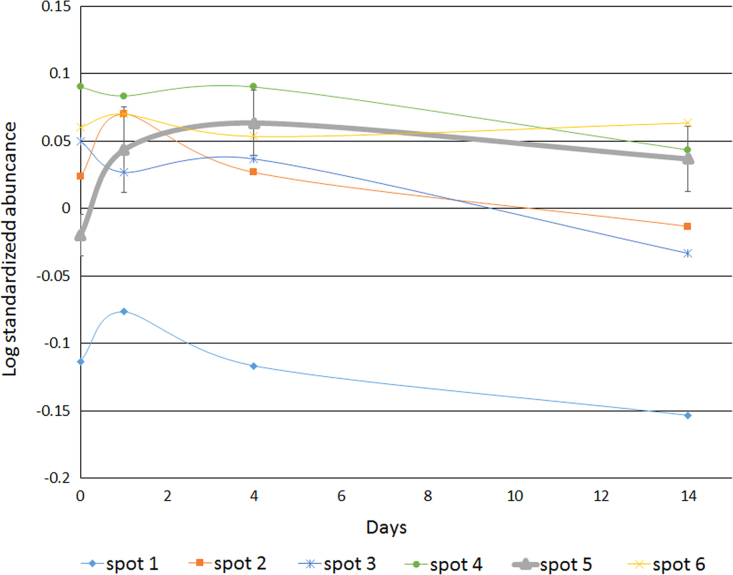
Dynamic abundance profiles of the root HSP70 spots 1, 2, 3, 4, 5 and 6 after 0, 1, 4 and 14 days of stress (n=3). For spot 1, 2, 3, 4 and 6 mean values are indicated. For spot 5 mean values ± SE are represented.

Meristem and root proteins were extracted and analyzed using the phenol extraction/ammonium acetate precipitation protocol reported by Carpentier et al. [10]. 50 µg of proteins was labeled with Cy2, Cy3 and Cy5 (GE Healthcare) for a total of 150 µg protein per gel, separated on gel and scanned according to Carpentier et al. [11]. Data were analyzed using the DeCyder software version 7.0 (GE Healthcare). Statistical analysis of the standardized abundance of spots was performed in DeCyder. Statistical analysis of the raw spot intensities was performed using ANOVA in STATISTICA software 10 on the log of the peak height of the internal standard samples exported from DeCyder. Dynamic abundance profiles of the root HSP70 spots 1, 2, 3, 4, 5 and 6 after 0, 1, 4 and 14 days of stress (*n*=3) can be found in Fig. 2.

For protein identification, gel pieces were extracted based on the protocol of Shevchenko et al. [12] for the in-gel reduction, alkylation and destaining of the proteins. The destaining step was performed twice after which the gel pieces were covered with 3 µL of 0.1 µg/µL trypsin and 47 µL trypsin buffer (25 µM ammonium carbonate, 10% acetonitrile (ACN)). Digestion was performed overnight at 37 °C. Peptides were extracted by adding 100 µL 5% ACN in 0.1% FA, vortexing, centrifuging and sonicating for 5 min after which the supernatant is removed to a new eppendorf tube. The whole peptide extraction process is repeated twice with 50 µL 10% ACN in 0.1% FA the first time and 50 µL 95% ACN and 5% FA the last time. The accumulated supernatant was then dried in a vacuum centrifuge and stored at −20 °C. Before analysis, the samples were resuspended in 0.1% FA and 5% ACN, desalted using C18 Zip Tips (Millipore) and eluted in 10 µl Milli-Q water with 0.1% FA and 60% ACN, dried in a vacuum centrifuge and resuspended in 0.1% FA and 5% ACN.

The HPLC-MS/MS analysis was performed on a Q Exactive Orbitrap mass spectrometer (Thermo Scientific, USA). The samples (5 µL) were injected and separated on an Ultimate 3000 HPLC system (Dionex, Thermo Scientific) equipped with a C18 PepMap100 precolumn (5 µm, 300 µm×5 mm, Thermo Scientific) and an EasySpray C18 column (3 µm, 75 µm×15 cm, Thermo Scientific) using a gradient of 5–20% ACN in 0.1% FA in 10 min followed by a gradient of 10–35% ACN in 0.1% FA in 4 min and then a final gradient from 35% to 95% ACN in 0.1% FA in 2.5 min. The flow-rate was set at 250 µL/min. The Q Exactive was operated in positive ion mode with a nanospray voltage of 1.5 kV and a source temperature of 250 °C. ProteoMAss LTQ/FT-Hybrid ESI Pos. Mode CalMix (MSCAL5-1EA SUPELCO, Sigma-Aldrich) was used as an external calibrant and the lock mass 445.12003 as an internal calibrant. The instrument was operated in data-dependent acquisition (DDA) mode with a survey MS scan at a resolution of 70,000 (fwhm at *m*/*z* 200) for the mass range of *m*/*z* 350–1800 for precursor ions, followed by MS/MS scans of the top 10 most intense peaks with +2, +3 and +4 charged ions above a threshold ion count of 16,000 at 35,000 resolution using normalized collision energy (NCE) of 28 eV with an isolation window of 3.0 *m*/*z* and dynamic exclusion of 10 s. All data were acquired with Xcalibur 2.2 software (Thermo Scientific). For identification, all raw data were converted into mgf files using Progenesis v4.1 (Nonlinear Dynamics, UK). The spectra were searched using Mascot (version 2.2.04) against our in-house *Musa* database (76,220 sequences) containing all the protein sequences of the published A and B genome plus contaminant sequences (trypsin and keratin). Redundancy was eliminated from the database using the program cdhit [13]. If both A and B isoforms were identical, the B genome isoform was eliminated. The original HSP70 protein sequences were removed and replaced by the manually curated HSP70 sequences. Search parameters were set at: tryptic digestion, one miscleavage allowed, 10 ppm precursor mass tolerance and 0.02 Da for fragment ion tolerance with a fixed modification of cysteine carbamidomethylation and a variable modification of methionine oxidation.

**Fig. 3:**
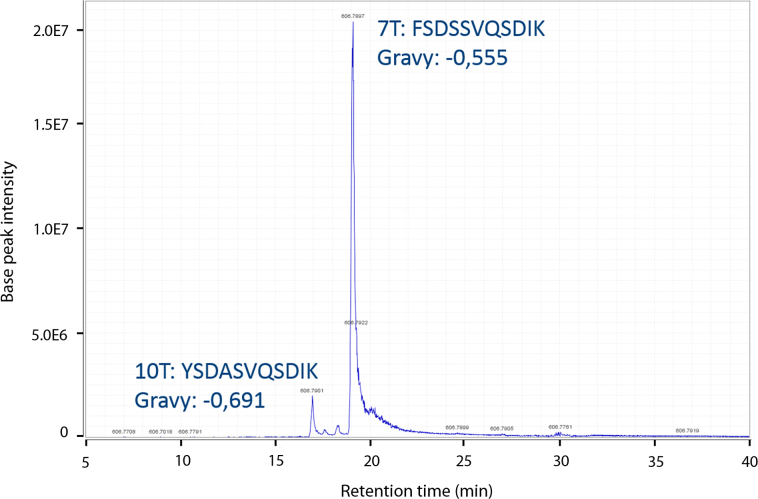
Differential retention time illustration of tryptic specific peptides from the HSP70 paralogs of chromosome 10 (YSDASVQSDIK) and of chromosome 7 (FSDSSVQSDIK). Although the peptides share an identical m/z (606.7908), their different amino acid constitution lead to different hydrophobic behavior as evidenced by the different GRAVY scores and therefore different retention times. GRAVY stands for grand average of hydropathicity and peptides with a more negative score are more hydrophilic and are eluted earlier during RP chromatography.

The advantage of an LC-separation is nicely illustrated by the separation of the peptides FSDSSVQSDIK (encoded by gene GSMUA_Achr7T15160) and YSDASVQSDIK (encoded by gene GSMUA_Achr10T00900). These two isoforms of the peptide have the same monoisotopic mass but have different retention times on the RP column because of their different hydrophobicity (approximately 19 and 17 min) (Fig. 3). This would have resulted in a chimeric spectrum using MALDI-TOF/TOF MS but produces separate spectra using LC-MS/MS.

An isoform was retained as positively identified in a spot if at least one tryptic specific peptide was found with an ion score higher than the Mascot identification score. Cytoscape v3.0 software was used to visualize tryptic specific peptides [14–16]. Supplementary file 3 contains a list of all identified HSP70 paralogs and allelic variants per spot based on this Mascot analysis. To quantify the different protein species in each spot, Mascot emPAI was exported and the ion intensity of the proteotypic peptide for each peptide was analyzed in Progenesis v4.1. Moreover, for all isoforms positively identified in at least one spot, we searched the unidentified MS/MS spectra in each spot in which they were not identified by performing a manual SRM approach. The ion intensity for a MS/MS spectrum was added to the quantification when the peptide fragment mass corresponded to the proteotypic peptide and a specific signature *m*/*z* was identified in the MS/MS spectrum. Several spots in a trail contain multiple proteins even on a 24 cm 3 pI zoom strip, as already been indicated by Schmidt et al. [17]. Although all the spots look well separated on the 2-DE gels they consist of several proteins caused by neighbor spots. The isoelectric focusing of one particular isoform is not restricted to one physical location in the gel and each isoform has its highest abundance at a particular isoelectric point (Supplementary file 4). Supplementary file 4 provides an overview of the ion intensity of the proteotypic peptide of each identified paralog and/or allelic variant in all spots.

## Footnotes

Appendix A Supplementary data associated with this article can be found in the online version at doi:10.1016/j.dib.2015.01.008.

## References

[1] Vanhove A., Vermaelen W., Swennen R., Carpentier S. (2015). A look behind the screens: Characterization of the HSP70 family during osmotic stress in a non-model crop. J. Proteomics.

[2] D’Hont A., Denoeud F., Aury J.-M., Baurens F.-C., Carreel F., Garsmeur O. (2012). The banana (*Musa acuminata*) genome and the evolution of monocotyledonous plants. Nature.

[3] Davey M.W., Gudimella R., Harikrishna J.A., Sin L.W., Khalid N., Keulemans J. (2013). A draft *Musa balbisiana* genome sequence for molecular genetics in polyploid, inter- and intra-specific Musa hybrids. BMC Genomics.

[4] G. Droc, D. Larivière, V. Guignon, N. Yahiaoui, D. This, O. Garsmeur, et al., The Banana Genome Hub, Database 2013, bat035 10.1093/database/bat035 PMC3662865 23707967

[5] Jung K.-H., Gho H.-J., Nguyen M., Kim S.-R., An G. (2013). Genome-wide expression analysis of HSP70 family genes in rice and identification of a cytosolic HSP70 gene highly induced under heat stress. Funct. Integr. Genomics.

[6] Larkin M.A., Blackshields G., Brown N.P., Chenna R., McGettigan P.A., McWilliam H. (2007). Clustal W and Clustal X version 2.0. Bioinformatics.

[7] Perrière G., Gouy M. (1996). WWW-query: an on-line retrieval system for biological sequence banks. Biochimie.

[8] Strosse H., Schoofs H., Panis B., Andre E., Reyniers K., Swennen R. (2006). Development of embryogenic cell suspensions from shoot meristematic tissue in bananas and plantains (Musa spp.). Plant Sci..

[9] Voets L., Dupré de Boulois H., Renard L., Strullu D.-G., Declerck S. (2005). Development of an autotrophic culture system for the *in vitro* mycorrhization of potato plantlets. FEMS Microbiol. Lett..

[10] Carpentier S., Witters E., Laukens K., Swennen R., Panis B. (2005). Two-dimensional gel electrophoresis and subsequent protein identification via MALDI-MS/MS; a successful approach to unravel the abiotic stress responses in a non-model organism (Musa spp.). Mol. Cell. Proteomics.

[11] Carpentier S.C., Swennen R., Panis B., Walker J.M. (2009). Plant protein sample preparation for 2DE. The Protein Protocols Handbook.

[12] Shevchenko A., Tomas H., Havlis J., Olsen J.V., Mann M. (2006). In-gel digestion for mass spectrometric characterization of proteins and proteomes. Nat. Protoc..

[13] Li W., Jaroszewski L., Godzik A. (2001). Clustering of highly homologous sequences to reduce the size of large protein databases. Bioinformatics.

[14] Shannon P., Markiel A., Ozier O., Baliga N.S., Wang J.T., Ramage D. (2003). Cytoscape: a software environment for integrated models of biomolecular interaction networks. Genome Res..

[15] Vertommen A., Moller A.L.B., Cordewener J.H.G., Swennen R., Panis B., Finnie C. (2011). A workflow for peptide-based proteomics in a poorly sequenced plant: a case study on the plasma membrane proteome of banana. J. Proteomics.

[16] Carpentier S., America T., Jorrin-Novo J.V., Komatsu S., Weckwerth W., Wienkoop S. (2014). Proteome analysis of orphan plant species, fact or fiction?. Plant Proteomics.

[17] Schmidt F., Schimd M., Jungblut P.R., Mattowb J., Faciusc A., Pleissner K. (2003). Iterative data analysis is the key for exhaustive analysis of peptide mass fingerprints from proteins separated by two-dimensional electrophoresis. J. Am. Soc. Mass Spectrom..

