# Supplementary material for: Data for the characterization of the HSP70 family during osmotic stress in banana, a non-model crop
Source: Data Brief. 2015 Feb 13;3:78–84. doi: 10.1016/j.dib.2015.01.008 (PMC4510055; doi:10.1016/j.dib.2015.01.008)
Supplement: Supplementary file 1 — Supplementary Data [file mmc1.zip › Supplementary file 3.pdf]

Supplementary File 3: Alignment of the main cytoplasmic HSP70 isoforms identified. The interdomain hinge is highlighted in yellow. The ATPase domain is surrounded by a green shape, the peptide binding domain by a blue shape and the C-terminal subdomain by an orange shape.

```
KMMuB_chr10_G28483          MAG-KGEGPAIGIDLGTTYSCVGVWQHDRVEIIANDQGNRTTPSYVAFTD 49
GSMUA_Achr10T00900_001      MAG-KGEGPAIGIDLGTTYSCVGVWQHDRVEIIANDQGNRTTPSYVAFTD 49
KMMuB_chr9_G25413           MAG-KGEGPAIGIDLGTTYSCVGVWQHDRVEIIANDQGNRTTPSYVGFTD 49
GSMUA_Achr9T03960_001       MAG-KGEGPAIGIDLGTTYSCVGVWQHDRVEIIANDQGNRTTPSYVGFTD 49
KMMuB_chr8_G23861           MAG-KGDGPAIGIDLGTTYSCVGVWQHDRVEIIANDQGNRTTPSYVAFTD 49
GSMUA_Achr8T20830_001       MAG-KGDGPAIGIDLGTTYSCVGVWQHDRVEIIANDQGNRTTPSYVAFTD 49
KMMuB_chr7_G19958           MAG-KGEGPAIGIDLGTTYSCVGVWQHDRVEIIANDQGNRTTPSYVAFTD 49
GSMUA_Achr7T15160_001       MAG-KGEGPAIGIDLGTTYSCVGVWQHDRVEIIANDQGNRTTPSYVAFTD 49
KMMuB_chr2_G04523           MAG-KGEGPAIGIDLGTTYSCVGVWQHDRVEIIANDQGNRTTPSYVAFTD 49
GSMUA_Achr2T16250_001       MAG-KGEGPAIGIDLGTTYSCVGVWQHDRVEIIANDQGNRTTPSYVAFTD 49
KMMuB_chr6_G18289           MAGGKGEGPAIGIDLGTTYSCVGVWQHDRVEIIANDQGNRTTPSYVAFTD 50
GSMUA_Achr6T34210_001       MAGGKGEGPAIGIDLGTTYSCVGVWQHDRVEIIANDQGNRTTPSYVAFTD 50
                            *** **:***************************************.***

KMMuB_chr10_G28483          TERLIGDAAKNQVAMNPTNTVFDAKRLIGRRYSDASVQSDIKLWPFKVVP 99
GSMUA_Achr10T00900_001      TERLIGDAAKNQVAMNPTNTVFDAKRLIGRRYSDASVQSDIKLWPFKVVP 99
KMMuB_chr9_G25413           TERLIGDAAKNQVAMNPTNTVFDAKRLIGRRFSDASVQSDIKLWPFKVIA 99
GSMUA_Achr9T03960_001       TERLIGDAAKNQVAMNPTNTVFDAKRLIGRRFSDASVQSDIKLWPFGVIA 99
KMMuB_chr8_G23861           TERLIGDAAKNQVAMNPINTVFDAKRLIGRRFTDASVQSDIKLWPFKVMS 99
GSMUA_Achr8T20830_001       TERLIGDAAKNQVAMNPINTVFDAKRLIGRRFTDASVQSDIKLWPFKVTS 99
KMMuB_chr7_G19958           SERLIGDAAKNQVAMNPINTVFDAKRLIGRRFSDSSVQSDIKLWPFKVIP 99
GSMUA_Achr7T15160_001       SERLIGDAAKNQVAMNPINTVFDAKRLIGRRFSDSSVQSDIKLWPFKVIP 99
KMMuB_chr2_G04523           TERLIGDAAKNQVAMNPINTVFDAKRLIGRRYSDPSVQSDIKLWPFKVIA 99
GSMUA_Achr2T16250_001       TERLIGDAAKNQVAMNPINTVFDAKRLIGRRYSDSSVQSDIKLWPFKVIA 99
KMMuB_chr6_G18289           TERLIGDAAKNQVAMNPINTVFDAKRLIGRRYSDPSVQSDIKLWPFKVIA 100
GSMUA_Achr6T34210_001       TERLIGDAAKNQVAMNPINTVFDAKRLIGRRYSDPSVQSDIKLWPFKVIA 100
                            :**************** *************::*.*********** * .

KMMuB_chr10_G28483          GPGDKPMIIVQYRGEEKQFSAEEISSMVLIKMKEIAEAYLGSTIKNAVVT 149
GSMUA_Achr10T00900_001      GPGDKPMIIVQYRGEDKQFSAEEISSMVLIKMKEIAEAYLGSTIKNAVVT 149
KMMuB_chr9_G25413           GPGDKPMIVVQYRGEEKQFAAEEISSMVLIKMKEIAEAYLGSTVKNAVVT 149
GSMUA_Achr9T03960_001       GPGDKPMIVVQYRGEEKQFAAEEISSMVLIKMKEIAEAYLGSTVKNAVVT 149
KMMuB_chr8_G23861           GPGDKPMIAVQYKGEEKQFSAEEISSMVLMKMKEIAEAYLGSVIKNAVVT 149
GSMUA_Achr8T20830_001       GPGDKPMIAVQYKGEEKQFSAEEISSMVLMKMKEIAEAYLGSVVKNAVVT 149
KMMuB_chr7_G19958           GPGDKPMIVVQYKGEDKQFAAEEISSMVLIKMKEIAEAYLGSVVKNAVVT 149
GSMUA_Achr7T15160_001       GPGDKPMIVVQYKGEDKQFAAEEISSMVLIKMKEIAEAYLGSVVKNAVVT 149
KMMuB_chr2_G04523           GPGDKPMIVVQYKGEEKQFSAEEISSMVLIKMREIAEAYLGTTIKNAVVT 149
GSMUA_Achr2T16250_001       GPGDKPMIVVQYKGEEKQFSAEEISSMVLIKMREIAEAYLGTTIKNAVVT 149
KMMuB_chr6_G18289           GPGDKPMIVVQYKGEEKQFSAEEISSMVLTKMKEIAEAYLGTTVKNAVVT 150
GSMUA_Achr6T34210_001       GPGDKPMIVVQYKGEEKQFSAEEISSMVLIKMKEIAEAYLGTTVKNAVVT 150
                            ******** ***:**:***:********* **:********:.:******

KMMuB_chr10_G28483          VPAYFNDSQRQATKDAGVIAGLNVMRIINEPTAAAIAYGLDKKASSVGEK 199
GSMUA_Achr10T00900_001      VPAYFNDSQRQATKDAGVIAGLNVMRIINEPTAAAIAYGLDKKASSVGEK 199
KMMuB_chr9_G25413           VPAYFNDSQRQATKDAGVIAGLNVMRIINEPTAAAIAYGLDKKASSVGEK 199
GSMUA_Achr9T03960_001       VPAYFNDSQRQATKDAGVIAGLNVMRIINEPTAAAIAYGLDKKVSSVGEK 199
KMMuB_chr8_G23861           VPAYFNDSQRQATKDAGVISGLNVMRIINEPTAAAIAYGLDKKASSVGEK 199
GSMUA_Achr8T20830_001       VPAYFNDSQRQATKDAGVISGLNVMRIINEPTAAAIAYGLDKKASSVGEK 199
KMMuB_chr7_G19958           VPAYFNDSQRQATKDAGVIAGLNVMRIINEPTAAAIAYGLDKKATSVGEK 199
GSMUA_Achr7T15160_001       VPAYFNDSQRQATKDAGVIAGLNVMRIINEPTAAAIAYGLDKKATSVGEK 199
KMMuB_chr2_G04523           VPAYFNDSQRQATKDAGVIAGLNVMRIINEPTAAAIAYGLDKKATSSGEK 199
GSMUA_Achr2T16250_001       VPAYFNDSQRQATKDAGVIAGLNVLRIINEPTAAAIAYGLDKKATTSGEK 199
KMMuB_chr6_G18289           VPAYFNDSQRQATKDAGVISGLNVMRIINEPTAAAIAYGLDKKASSSGEK 200
GSMUA_Achr6T34210_001       VPAYFNDSQRQATKDAGVISGLNVMRIINEPTAAAIAYGLDKKASSSGEK 200
                            *******************:****:******************.:: ***

KMMuB_chr10_G28483          NVLIFDLGGGTFDVSLLTIEEGIFEVKATAGDTHLGGEDFDNRMVNHFVQ 249
GSMUA_Achr10T00900_001      NVLIFDLGGGTFDVSLLTIEEGIFEVKATAGDTHLGGEDFDNRMVNHFVQ 249
KMMuB_chr9_G25413           NVLIFDLGGGTFDVSLLTIEEGIFEVKATAGDTHLGGEDFDNRMVNHFVQ 249
GSMUA_Achr9T03960_001       NVLIFDLGGGTFDVSLLTIEEGIFEVKATAGDTHLGGEDFDNRMVNHFVQ 249
KMMuB_chr8_G23861           NVLIFDLGGGTFDVSLLTIEEGIFEVKATAGDTHLGGEDFDNRMVNHFVQ 249
GSMUA_Achr8T20830_001       NVLIFDLGGGTFDVSLLTIEEGIFEVKATAGDTHLGGEDFDNRMVNHFVQ 249
KMMuB_chr7_G19958           NVLIFDLGGGTFDVSLLTIEEGIFEVKATAGDTHLGGEDFDNRMVNHFVQ 249
GSMUA_Achr7T15160_001       NVLIFDLGGGTFDVSLLTIEEGIFEVKATAGDTHLGGEDFDNRMVNHFVQ 249
KMMuB_chr2_G04523           NVLIFDLGGGTFDVSLLTIEEGIFEVKATAGDTHLGGEDFDNRMVNHFVQ 249
GSMUA_Achr2T16250_001       NVLIFDLGGGTFDVSLLTIEEGIFEVKATAGDTHLGGEDFDNRMVNHFVQ 249
KMMuB_chr6_G18289           NVLIFDLGGGTFDVSLLTIEEGIFEVKATAGDTHLGGEDFDNRMVNHFVQ 250
GSMUA_Achr6T34210_001       NVLIFDLGGGTFDVSLLTIEEGIFEVKATAGDTHLGGEDFDNRMVNHFVQ 250
                            **************************************************
```

```
KMMuB_chr10_G28483          EFKRKNKKDISGNPRALRRLRTACERAKRTLSSTAQTTIEIDSLYEGIDF 299
GSMUA_Achr10T00900_001      EFKRKNKKDISGNPRALRRLRTACERAKRTLSSTAQTTIEIDSLYEGIDF 299
KMMuB_chr9_G25413           EFKRKHKKDISGNPRALRRLRTACERAKRTLSSTAQTTIEIDSLYEGVDF 299
GSMUA_Achr9T03960_001       EFKRKHKKDISGNPRALRRLRTACERAKRTLSSTAQTTIEIDSLYEGVDF 299
KMMuB_chr8_G23861           EFKRKHKKDISGNPRALRRLRTACERAKRTLSSTAQTTIEIDSLYEGVDF 299
GSMUA_Achr8T20830_001       EFKRKHKKDISGNPRALRRLRTACERAKRTLSSTAQTTIEIDSLYEGVDF 299
KMMuB_chr7_G19958           EFKRKNKKDISGNPRALRRLRTACERAKRTLSSTAQTTIEIDSLYEGIDF 299
GSMUA_Achr7T15160_001       EFKRKNKKDISGNPRALRRLRTACERAKRTLSSTAQTTIEIDSLYEGIDF 299
KMMuB_chr2_G04523           EFKRKHKKDISGNPRALRRLRTACERAKRTLSSTAQTTIEIDSLYEGIDF 299
GSMUA_Achr2T16250_001       EFKRKHKKDISGNPRALRRLRTACERAKRTLSSTAQTTIEIDSLYEGIDF 299
KMMuB_chr6_G18289           EFKRKHKKDISGNPRALRRLRTACERAKRTLSSTAQTTIEIDSLFEGIDF 300
GSMUA_Achr6T34210_001       EFKRKHKKDISGNPRALRRLRTACERAKRTLSSTAQTTIEIDSLFEGIDF 300
                            *****:**************************************:**:**

KMMuB_chr10_G28483          YSTITRARFEELNMDLFRKCMEPVEKCLRDAKMDKSSVHDVVLVGGSTRI 349
GSMUA_Achr10T00900_001      YSTITRARFEELNMDLFRKCMEPVEKCLRDAKMDKSSVHDVVLVGGSTRI 349
KMMuB_chr9_G25413           YTTITRARFEELNMDLFRKCMEPVEKCLRDAKMDKSSVHDVVLVGGSTRI 349
GSMUA_Achr9T03960_001       YTTITRARFEELNMDLFRKCMEPVEKCLKDAKMDKSSVHDVVLVGGSTRI 349
KMMuB_chr8_G23861           YTTITRARFEELNMDLFRKCMDPVEKCLRDAKMDKSSVDDVVLVGGSTRI 349
GSMUA_Achr8T20830_001       YTTITRARFEELNMDLFRKCMDPVEKCLRDAKMDKSSVDDVVLVGGSTRI 349
KMMuB_chr7_G19958           YSTITRARFEELNMDLFRKCMEPVEKCLRDAKMDKSTVHDVVLVGGSTRI 349
GSMUA_Achr7T15160_001       YSTITRARFEELNMDLFRKCMEPVEKCLRDAKMDKSTVHDVVLVGGSTRI 349
KMMuB_chr2_G04523           YSTITRARFEELNMDLFRKCMEPVEKCLRDAKMDKSSIHDVVLVGGSTRI 349
GSMUA_Achr2T16250_001       YSTITRARFEELNMDLFRKCMEPVEKCLRDAKMDKSSIHDVVLVGGSTRI 349
KMMuB_chr6_G18289           YSTITRARFEELNMDLFRKCMEPVEKCLRDAKMDKSSVHDVVLVGGSTRI 350
GSMUA_Achr6T34210_001       YSTITRARFEELNMDLFRKCMEPVEKCLRDAKMDKSSVHDVVLVGGSTRI 350
                            *:*******************:******:*******::.***********

KMMuB_chr10_G28483          PKVQQLLQDFFNGKELCKSINPDEAVAYGAAVQAAILSGEGNEKVQDLLL 399
GSMUA_Achr10T00900_001      PKVQQLLQDFFNGKELCKSINPDEAVAYGAAVQAAILSGEGNEKVQDLLL 399
KMMuB_chr9_G25413           PKVQQLLQDFFNGKELCKSINPDEAVAYGAAVQAAILSGEGNEKVQDLLL 399
GSMUA_Achr9T03960_001       PKVQQLLQDFFNGKELCKSINPDEAVAYGAAVQAAILSGEGNEKVQDLLL 399
KMMuB_chr8_G23861           PKVQQLLQDFFNGKELCKSINPDEAVAYGAAVQAAILSGEGNEKVQDLLL 399
GSMUA_Achr8T20830_001       PKVQQLLQDFFNGKELCKSINPDEAVAYGAAVQAAILSGEGNEKVQDLLL 399
KMMuB_chr7_G19958           PKVQQLLQDFFNGKELCKSINPDEAVAYGAAVQAAILSGEGNEKVQDLLL 399
GSMUA_Achr7T15160_001       PKVQQLLQDFFNGKELCKSINPDEAVAYGAAVQAAILSGEGNEKVQDLLL 399
KMMuB_chr2_G04523           PKVQQLLQDFFNGKELCKSINPDEAVAYGAAVQAAILSGEGNEKVQDLLL 399
GSMUA_Achr2T16250_001       PKVQQLLQDFFNGKELCKSINPDEAVAYGAAVQAAILSGEGNEKVQDLLL 399
KMMuB_chr6_G18289           PKVQQLLQDFFNGKELCKSINPDEAVAYGAAVQAAILSGEGNEKVQDLLL 400
GSMUA_Achr6T34210_001       PRVQQLLQDFFNGKELCKSINPDEAVAYGAAVQAAILSGEGNEKVQDLLL 400
                            *:************************************************

KMMuB_chr10_G28483          LDVTPLSLGLETAGGVMTVLIPRNTTIPTKKEQVFSTYSDNQPGVLIQVY 449
GSMUA_Achr10T00900_001      LDVTPLSLGLETAGGVMTVLIPRNTTIPTKKEQVFSTYSDNQPGVLIQVY 449
KMMuB_chr9_G25413           LDVTPLSLGLETAGGVMTVLIPRNTTIPTKKEQVFSTYSDNQPGVLIQVY 449
GSMUA_Achr9T03960_001       LDVTPLSLGLETAGGVMTVLIPRNTTIPTKKEQVFSTYSDNQPGVLIQVY 449
KMMuB_chr8_G23861           LDVTPLSLGLETAGGVMTVLIPRNTTIPTKKEQVFSTYSDNQPGVLIQVY 449
GSMUA_Achr8T20830_001       LDVTPLSLGLETAGGVMTVLIPRNTTIPTKKEQVFSTYSDNQPGVLIQVY 449
KMMuB_chr7_G19958           LDVTPLSLGLETAGGVMTVLIPRNTTIPTKKEQVFSTYSDNQPGVLIQVY 449
GSMUA_Achr7T15160_001       LDVTPLSLGLETAGGVMTVLIPRNTTIPTKKEQVFSTYSDNQPGVLIQVY 449
KMMuB_chr2_G04523           LDVTPLSLGLETAGGVMTVLIPRNTTIPTKKEQVFSTYSDNQPGVLIQVY 449
GSMUA_Achr2T16250_001       LDVTPLSLGLETAGGVMTVLIPRNTTIPTKKEQVFSTYSDNQPGVLIQVY 449
KMMuB_chr6_G18289           LDVTPLSLGLETAGGVMTVLIPRNTTIPTKKEQVFSTYSDNQPGVLIQVY 450
GSMUA_Achr6T34210_001       LDVTPLSLGLETAGGVMTVLIPRNTTIPTKKEQVFSTYSDNQPGVLIQVY 450
                            **************************************************

KMMuB_chr10_G28483          EGERTRTRDNN-LLGKFELSGIPPAPRGVPQITVCFDIDANGILNVSAED 498
GSMUA_Achr10T00900_001      EGERTRTRDNN-LLGKFELSGIPPAPRGVPQITVCFDIDANGILNVSAED 498
KMMuB_chr9_G25413           EGERTRTRDNN-LLGKFELSGIPPAPRGVPQITVCFDIDANGILNVSAED 498
GSMUA_Achr9T03960_001       EGERTRTRDNNNLLGKFELSGIPPAPRGVPQITVSFDIDANGILNVSAED 499
KMMuB_chr8_G23861           EGERTRTRDNN-LLGKFELSGIPPAPRGVPQITVCFDIDANGILNVSAED 498
GSMUA_Achr8T20830_001       EGERTRTRDNN-LLGKFELSGIPPAPRGVPQITVCFDIDANGILNVSAED 498
KMMuB_chr7_G19958           EGERTRTRDNN-LLGKFELSGIPPAPRGVPQITVCFDIDANGILNVSAED 498
GSMUA_Achr7T15160_001       EGERTRTRDNN-LLGKFELSGIPPAPRGVPQITVCFDIDANGILNVSAED 498
KMMuB_chr2_G04523           EGERTRTRDNN-LLGKFELSGIPPAPRGVPQITVCFDIDANGILNVSAED 498
GSMUA_Achr2T16250_001       EGERTRTRDNN-LLGKFELSGIPPAPRGVPQITVCFDIDANGIINVSAED 498
KMMuB_chr6_G18289           EGERARTKDNN-LLGKFELSGIPPAPRGVPQITVCFDIDANGILNVSAED 499
GSMUA_Achr6T34210_001       EGERARTKDNN-LLGKFELSGIPPAPRGVPQITVCFDIDANGILNVSAED 499
                            ****:**:*** **********************.********:******
```

```
KMMuB_chr10_G28483         KTTGQKNKITITNDKGRLSKEEIENMVQEAEKYKAEDEEHKKKVEAKNAL 548
GSMUA_Achr10T00900_001     KTTGQKNKITITNDKGRLSKEEIENMVQEAEKYKAEDEEHKKKVEAKNAL 548
KMMuB_chr9_G25413          KTTGQKNKITITNDKGRLSKEEIENMVQEAEKYKAEDEEHKKKVEAKNAL 548
GSMUA_Achr9T03960_001      KTTGQKNKITITNDKGRLSKEEIENMVQEAEKYKAEDEEHKKKVEAKNAL 549
KMMuB_chr8_G23861          KTTGQKNKITITNDKGRLSKEDIEKMVQEAEKYKSEDEEHKKKVEAKNAL 548
GSMUA_Achr8T20830_001      KTTGQKNKITITNDKGRLSKEDIEKMVQEAEKYKSEDEEHKKKVEAKNAL 548
KMMuB_chr7_G19958          KTTGQKNKITITNDKGRLSKEEIEKMVQEAEKYKSEDEEHKKKVESKNAL 548
GSMUA_Achr7T15160_001      KTTGQKNKITITNDKGRLSKEEIEKMVQEAEKYKSEDEEHKKKVESKNAL 548
KMMuB_chr2_G04523          KTTGQKNKITITNDKGRLSKGEIEKMVQEAEKYKAEDEEHKKKIEAKNAL 548
GSMUA_Achr2T16250_001      KTTGQKNKITVTNDKGRLSKGEIEKMVQEAEKYKGEDEEHKKKIEAKNAL 548
KMMuB_chr6_G18289          KTTGQKNKITITNDKGRLSKEEIEKMVQEAEKYKAEDEDHKKKVEAKNAL 549
GSMUA_Achr6T34210_001      KTTGQKNKITITNDKGRLSKEEIEKMVQEAEKYKAEDEDHKKKVEAKNAL 549
                           **********:********* :**:*********.***:****:*:****

KMMuB_chr10_G28483         ENYAYNMRNTIKDDKIASKLAAADRKKIEDAIEQAIQWLDGNQLAEADEF 598
GSMUA_Achr10T00900_001     ENYAYNMRNTIKDDKIASKLAAADRKKIEDAIEQAIQWLDGNQLAEADEF 598
KMMuB_chr9_G25413          ENYAYNMRNTIKDEKIASKLADADKKKIEDAIEQAIQWLDGNQLAEADEF 598
GSMUA_Achr9T03960_001      ENYAYNMRNTIKDEKIASKLADADKKKIEDAIEQAIQWLNGNQLAEADEF 599
KMMuB_chr8_G23861          ENYSYNMRNTIKDDKIASKLAPADKKKIEDAIDQAIQWLDGNQLAEADEF 598
GSMUA_Achr8T20830_001      ENYSYNMRNTIKDDKIASKLAAADKKKIEDAIDQAIQWLDGNQLAEADEF 598
KMMuB_chr7_G19958          ENYAYNMRNTINDDKIASKLAAADKKKIEDAIEQAIQWLDGNQLAEADEF 598
GSMUA_Achr7T15160_001      ENYAYNMRNTINDDKIASKLAAADKKKIEDAIEQAIQWLDGNQLAEADEF 598
KMMuB_chr2_G04523          ENYAYNMRNTIRDEKIAAKLPAEDKKKIEDAVEQAISWLDGNQLAEADEF 598
GSMUA_Achr2T16250_001      ENYSYNMRNTIRDEKIAAKLPAEDKKKIEDAVEQAISWLDGNQLAEADEF 598
KMMuB_chr6_G18289          ENYAYNMRNTIKDEKIAAKLPAADKKKIEDAIEKAISWLDGNQLAEADEF 599
GSMUA_Achr6T34210_001      ENYAYNMRNTIKDEKIAAKLPAADKKKIEDAIEKAISWLDGNQLAEADEF 599
                           ***:*******.*:***:**.  *:******:::**.**:**********

KMMuB_chr10_G28483         EDKMKELESI[illegible]NPIIAKMYQGAGADMAGGMDDDVPSAGGSG-AGPKIEEV 647
GSMUA_Achr10T00900_001     EDKMKELESI[illegible]NPIIAKMYQGAGADMAGEMDDDVPAAGGSG-AGPKIEEV 647
KMMuB_chr9_G25413          EDKMKELESI[illegible]NPIIAKMYQGAGADMAGGMDDDVPSAGGSG-TGPKIEDQ 647
GSMUA_Achr9T03960_001      EDKMKELESI[illegible]NPIIAKMYQGAGADMAGRMDDDVPSAGGSG-TGPKIEDQ 648
KMMuB_chr8_G23861          EDKMKELESI[illegible]NPIIAKMYQGAGADMGGEMDDDAPSAGASG-AGPKIEEV 647
GSMUA_Achr8T20830_001      EDKMKELESI[illegible]NPIIAKMYQGAGADMGGGMDDDAPSAGASG-AGPKIEEV 647
KMMuB_chr7_G19958          DDKMKELESI[illegible]NPIIAKMYQGAGADMAGGMDDDAPPAGGSG-AGPKIEEV 647
GSMUA_Achr7T15160_001      DDKMKELESI[illegible]NPIIAKMYQGAGADMAGGMDDDAPPAGGSG-AGPKIEEV 647
KMMuB_chr2_G04523          EDRMKELESI[illegible]NPIIAKMYQGAGGGTGGGMDEDIPSTGGSSGAGPKIEEV 648
GSMUA_Achr2T16250_001      EDRMKELESI[illegible]NPIIAKMYQGAGGGMGGGMDEDIPSTGGSSGAGPKIEEV 648
KMMuB_chr6_G18289          EDKMKELEGI[illegible]NPIIAKMYQGAGADMAGGMDEDGPTTGGSS-AGPKIEEV 648
GSMUA_Achr6T34210_001      EDKMKELEGI[illegible]NPIIAKMYQGAGADMAGGMDEDGPTTGGSS-AGPKIEEV 648
                           :*:*****.**************..  .* **:* *.:*.*. :*****:

KMMuB_chr10_G28483         D------------------------------------------------- 648
GSMUA_Achr10T00900_001     D------------------------------------------------- 648
KMMuB_chr9_G25413          LVAVVLGRRLRRLIKCDHSVLSAGCFDSRGVVLVWYHRYVVLGLLAP--- 694
GSMUA_Achr9T03960_001      LVAVVLGRRLRRLIKCDHSVLSAGCFDSRGVVLVWYQRYVVLDFWPHEMG 698
KMMuB_chr8_G23861          D------------------------------------------------- 648
GSMUA_Achr8T20830_001      D------------------------------------------------- 648
KMMuB_chr7_G19958          D------------------------------------------------- 648
GSMUA_Achr7T15160_001      D------------------------------------------------- 648
KMMuB_chr2_G04523          D------------------------------------------------- 649
GSMUA_Achr2T16250_001      D------------------------------------------------- 649
KMMuB_chr6_G18289          D------------------------------------------------- 649
GSMUA_Achr6T34210_001      D------------------------------------------------- 649
```
